# Effect of graphene flake size on functionalisation: quantifying reaction extent and imaging locus with single Pt atom tags†

**DOI:** 10.1039/d1sc01958a

**Published:** 2021-10-21

**Authors:** Noelia Rubio, Heather Au, Gabriel O. Coulter, Laure Guetaz, Gerard Gebel, Cecilia Mattevi, Milo S. P. Shaffer

**Affiliations:** Departments of Chemistry & Materials, Imperial College London London UK m.shaffer@imperial.ac.uk; Department of Chemical Engineering, Imperial College London London UK; University Grenoble Alpes, CEA, LITEN 38054 Grenoble Cedex 9 France; Department of Materials, Imperial College London London UK

## Abstract

Here, the locus of functionalisation on graphene-related materials and the progress of the reaction is shown to depend strongly on the starting feedstock. Five characteristically different graphite sources were exfoliated and functionalized using a non-destructive chemical reduction method. These archetypical examples were compared *via* a model reaction, grafting dodecyl addends, evaluated with TGA-MS, XPS and Raman data. A general increase in grafting ratio (ranging from 1.1 wt% up to 25 wt%) and an improvement in grafting stoichiometry (C/R) were observed as flake radius decreased. Raman spectrum imaging of the functionalised natural flake graphite identified that grafting is directed towards flake edges. This behaviour was further corroborated, at atomistic resolution, by functionalising the graphene layers with bipyridine groups able to complex single platinum atoms. The distribution of these groups was then directly imaged using aberration-corrected HAADF-STEM. Platinum atoms were found to be homogeneously distributed across smaller graphenes; in contrast, a more heterogeneous distribution, with a predominance of edge grafting was observed for larger graphites. These observations show that grafting is directed towards flake edges, but not necessary at edge sites; the mechanism is attributed to the relative inaccessibility of the inner basal plane to reactive moieties, resulting in kinetically driven grafting nearer flake edges. This phenomenology may be relevant to a wide range of reactions on graphenes and other 2d materials.

## Abstract 2

The flake size and morphology of the starting material strongly effect the degree and location of grafting when using reductive functionalisation.

## Graphical abstract

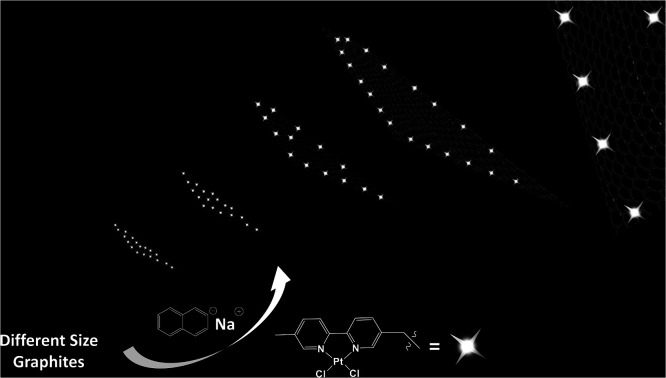

## 1. Introduction

Graphene has attracted attention in a wide range of scientific fields and applications. Covalent modification of graphene is useful both to assist processing, for example by raising solubility or increasing exfoliation, and to introduce functions, such as doping, analyte binding, or fluorescence. Reductive functionalisation methods have been applied to many different systems and grafting moieties, due to their versatility and ability to preserve the framework structure.^1^ However, there is still limited understanding of many crucial factors that govern the reaction outcomes; the many different methodologies (starting materials, charging ratios, metals, solvents, grafting moieties) make it hard to unify results. Different graphite sources vary widely in terms of morphological and physical properties,^2^ affecting the efficiency, extent, and locus of the functionalization reaction.^3^ The different reactivity between graphite sources (natural, powder and spherical graphite) during reductive alkylation has been studied^1*b*^ but systematic trends between grafting ratios and flake size were not explored. In graphene reactions more generally, it has been observed that single layer graphene (SLG) has a higher reactivity than bi/tri layer graphene (BLG/TLG) and bulk graphite.^4^ A more fundamental unanswered question in graphene chemistry relates to the distribution of the grafted groups, whether they correlate with edges, point defects, or each other. Grafting is sometimes inhomogeneous, starting from the edges and/or forming islands or clusters.^3,4^ The spatial distributions of the functional groups will determine the physical and chemical properties of the functionalised material but are challenging to determine and harder to control.^3,5^ Indeed, the literature contains a number of conflicting arguments, in part arising from the need to rely on indirect techniques to determine the reaction sites. Grafting is usually demonstrated by bulk methods, such as thermogravimetric analysis coupled with mass spectrometry (TGA-MS) or Raman spectroscopy. However, TGA only indicates the overall proportion of the grafted species whilst the interpretation of Raman spectroscopy usually assumes homogeneous grafting and at best provides only low resolution mapping. There are rare examples of atomic-resolution quantification and mapping techniques applied to the study of nanocarbon chemistry, using high-resolution microscopies. For example, high-angle annular dark-field (HAADF)/scanning electron microscopy (STEM) has been used to study the distribution of heavy atoms complexed to graphene oxide (GO) functional groups,^6^ whilst STEM-EELS (energy electron loss spectroscopy) was used to study the distribution of oxygen containing groups on carbon nanotubes.^7^ Scanning probe microscopy techniques, such as scanning tunnelling microscopy (STM), can resolve defects, particularly on clean well defined SLGs, and have been used to map the distribution of both grafted organic groups and metals.^8,9^

Here, we perform a systematic study of the reactivity of a range of graphene feedstocks under reductive conditions, using atomic resolution tagging to identify the patterns of functionalisation. Five graphite sources are exfoliated and functionalised with dodecyl chains using an archetypical reductive chemistry.^10^ This technique allows the formation of exfoliated charged graphene layers (“graphenides”) that can be further functionalised with tailored functional groups for specific application. The graphitic sources were selected to represent examples of common feedstocks, including different flake sizes and degrees of crystallinity. Grafting ratios, morphology and structural properties were directly correlated to the flake size, and the nature of the reaction explored indirectly with Raman spectroscopy. In order to visualise the distribution of functional groups directly, these graphene-related materials were functionalized with a bipyridine group that can coordinate individual platinum atoms, thus making the functionalization sites visible with aberration-corrected transmission electron microscopy in HAADF-STEM mode. As well as directly tagging the pattern of functionalization, bound platinum atoms represent a potentially exciting class of supported catalysts.

## 2. Experimental section

### 2.1 Materials

Large flake natural graphite was purchased from NGS Naturgraphit GmbH with a purity of 99.9% C and flake size of 2.5 mm, stated by the manufacturer. Natural flake graphite (NFG) was obtained from Graphexel Ltd (grade: 2369). Graphite nanoplatelets (Elicarb Materials Grade Graphene Powder SP8082) were supplied by Thomas Swan, with a typical lateral size of 1–5 μm and carbon content of 98%. Few-layer graphene (FLG) was obtained from CamGraph nanosystems UK, with a carbon purity >99.5% and an average lateral size of 0.5 μm. Graphite nanofibers (GNF) were supplied by Future Carbon GmbH (batch no. A09-122.123). All the graphite starting materials were used without any further purification, though dried thoroughly as described below. Tetrahydrofuran, dried in-house in a solvent-drying tower packed with alumina, and dodecyl bromide (Sigma-Aldrich) were degassed *via* a freeze–pump–thaw method then further dried over 20 vol% 4 Å activated molecular sieves. Naphthalene (99%, Sigma-Aldrich) was dried under vacuum in the presence of phosphorus pentoxide before use. Sodium (99.95%, ingot) and potassium hexachloroplatinate were purchased from Sigma-Aldrich and used as received. 5-Monobromomethyl-5′-methyl-2,2′-bipyridine (**1**) was synthesised according to a procedure described in literature.^12^

### 2.2 Experimental procedures

#### Preparation of sodium-naphthalide solution

A stock sodium-naphthalide solution was prepared to allow for accurate, simple addition of sodium to the corresponding graphite starting material. 23 mg (1 mmol) sodium and 128 mg (1 mmol) dried naphthalene were added to 10 mL degassed anhydrous THF in a N_2_-filled glove box, and stirred for 1 day until all sodium had dissolved, forming a dark-green solution.

#### Synthesis of Na-THF-graphite

A Young's tube containing graphite starting material (15 mg, 1.25 mmol carbon) and a magnetic stirrer bar was heated at 400 °C for 1 h under vacuum, and then kept under vacuum for 16 h at room temperature, before placing in a glove box. 1.04 mL of sodium-naphthalide solution was added to the Young's tube and the concentration of graphite in THF adjusted to 0.1 M by addition of 11.46 mL of THF (C/Na = 12, [Na] = 0.008 M). The suspension was stirred at room temperature for 1 day under N_2_. To quench the product, dry O_2_/N_2_ (20/80%, ∼1 L) was bubbled into the solution for 15 min, then stirred overnight under dry O_2_/N_2_ to quench any remaining charges. The mixture was filtered through a 0.1 μm PTFE membrane and washed thoroughly with THF, ethanol and water to remove any residual naphthalene and sodium salts formed during the reaction. The product was obtained as a dark powder after washing with ethanol and drying overnight under vacuum at 80 °C.

#### Synthesis of C_12_H_25_-graphite

A Young's tube containing graphite starting material (15 mg, 1.25 mmol carbon) and a magnetic stirrer bar was heated at 400 °C for 1 h under vacuum, and then kept under vacuum for 16 h at room temperature, before placing in a glove box. 1.04 mL of sodium-naphthalide solution was added to the Young's tube and the concentration of graphite in THF adjusted to 0.1 M by addition of 11.46 mL of THF (C/Na = 12, [Na] = 0.008 M). 1-Bromododecane (0.31 mmol, 77 mg, 1 : 3 ratio Na : dodecyl bromide) was subsequently added to the suspension and the reaction was stirred for one day at room temperature. To quench the product, dry O_2_/N_2_ (20/80%, ∼1 L) was bubbled into the solution for 15 min, then stirred overnight under dry O_2_/N_2_ to quench any remaining charges. The mixture was filtered through a 0.1 μm PTFE membrane and washed thoroughly with THF, hexane, ethanol and water to remove any residual naphthalene, unreacted 1-bromododecane and sodium salts formed during the reaction. The product was obtained as a dark powder after washing with ethanol and drying overnight under vacuum at 80 °C.

#### Preparation of dodecane adsorption controls

In an identical procedure to above, dried and degassed dodecane (0.071 mL, 0.313 mmol), in place of 1-bromododecane, was added dropwise to the graphenide dispersion. After stirring at room temperature for 1 day under nitrogen, the reaction was quenched with dry O_2_/N_2_, and washed and dried as before, to yield the final product.

#### Synthesis of bipyridine-graphite

A Young's tube containing graphite starting material (15 mg, 1.25 mmol carbon) and a magnetic stirrer bar was heated at 400 °C for 1 h under vacuum, and then kept under vacuum for 16 h at room temperature, before placing in a glove box. 1.04 mL of sodium-naphthalide solution was added to the Young's tube and the concentration of graphite in THF adjusted to 0.1 M by addition of 11.46 mL of THF (C/Na = 12, [Na] = 0.008 M). 5-Monobromomethyl-5′-methyl-2,2′-bipyridine (**1**) (0.31 mmol, 80 mg, 1 : 3 ratio Na : (**1**)) was subsequently added to the suspension and the reaction was stirred for one day at room temperature. To quench the product, dry O_2_/N_2_ (20/80%, ∼1 L) was bubbled into the solution for 15 min, and then stirred overnight under dry O_2_/N_2_ to quench any remaining charges. The mixture was filtered through a 0.1 μm PTFE membrane and washed thoroughly with THF, hexane, ethanol and water to remove any residual naphthalene, unreacted reagent, bromide and sodium salts formed during the reaction. The product was obtained as a dark powder after washing with ethanol and drying overnight under vacuum at 80 °C.

#### Synthesis of Pt-bipyridine-graphite

A round bottom flask containing bipy-graphite (20 mg) suspended in aqueous HCl (2 M, 20 mL) was sonicated for 5 minutes and K_2_PtCl_6_ (0.021 mmol, 10 mg) was subsequently added. The mixture was refluxed at 100 °C for 3 h. After stirring overnight at room temperature, the product was filtered through a 0.1 μm PTFE membrane and washed thoroughly with water and ethanol. The product was obtained as a dark powder after washing with ethanol and drying overnight under vacuum at 80 °C.

### 2.3 Equipment and characterisation

Thermogravimetric analysis coupled with mass spectrometry (TGA-MS) was performed using a Mettler Toledo TGA/DSC 1 instrument integrated with a Hiden HPR-20 QIC EGA mass spectrometer under nitrogen atmosphere. Samples were held at 100 °C for 30 min, then heated from 100 °C to 850 °C at 10 °C min^−1^ (N_2_ flow rate = 60 mL min^−1^). X-ray photoelectron spectroscopy (XPS) data were recorded using a K-alpha^+^ XPS spectrometer equipped with an MXR3 Al Kα monochromated X-ray source (*hν* = 1486.6 eV). X-ray gun power was set to 72 W (6 mA and 12 kV). Charge compensation was achieved with the FG03 flood gun using a combination of low energy electrons and the ion flood source. Survey scans were acquired using 200 eV pass energy, 1 eV step size and 100 ms (50 ms × 2 scans) dwell times. All high resolution spectra were acquired using 20 eV pass energy, 0.1 eV step size and 1 s (50 ms × 20 scans) dwell times; pressure was ≤1 × 10^−8^ mbar. Measured signals were stable during continued data acquisition and did not vary on the timescale of the experiment. Samples were prepared by pressing the sample onto carbon-based double-sided tape. Atomic compositions were calculated from averaged spectra obtained from at least 3 areas per sample. Raman spectra were collected on a Renishaw inVia micro-Raman (1000–3000 cm^−1^), using a 50 mW 532 nm laser at 10% laser power. Statistical Raman data were obtained from measurements carried out in streamline mode of at least 500 spectra per sample, from three independent areas. Samples were prepared by drop casting dispersions on a glass slide or silicon wafer. For transmission electron microscopy analyses, samples were prepared by drop-casting dilute graphene dispersions onto a on lacey carbon coated Cu TEM grids (Agar Scientific). Analyses were performed in HAADF/STEM mode using a FEI-Titan Themis microscope equipped with a probe corrector and working at 200 kV. Images were recorded rapidly to avoid potential sample degradation; the Pt atom distribution did not change during sample imaging. Transmission Electron Microscopy (TEM) was carried out using a JEOL 2100Plus TEM at 200 kV operating voltage. Ambient XRD data was recorded on a PANalytical X'Pert PRO diffractometer operating at 40 kV and 40 mA, with CuK_α_ (*λ* = 1.542 Å) radiation, at a scan rate of 0.085° s^−1^, step size of 0.0334°, and 2*θ* varying between 5° and 60°. Dried powder samples (510 mg) were mounted onto a zero-background Si sample holder (PANalytical Ltd, UK) and levelled to the height of the top of the holder using a glass slide. SEM images were taken using a Leo Gemini 1525 field emission gun scanning electron microscope (FEGSEM) with SmartSEM software, at an accelerating voltage of 5 keV, working distance of ∼7 mm and a 30 μm aperture. Powder samples were fixed onto aluminium stubs using carbon tabs (Agar Scientific Ltd). Tapping-mode atomic force microscopy measurements were taken using Bruker MultiMode 8 AFM. Samples for AFM were prepared by drop-casting dilute dispersed-graphene chloroform solutions on silica substrates.

## 3. Results and discussion

**Fig. 1:**
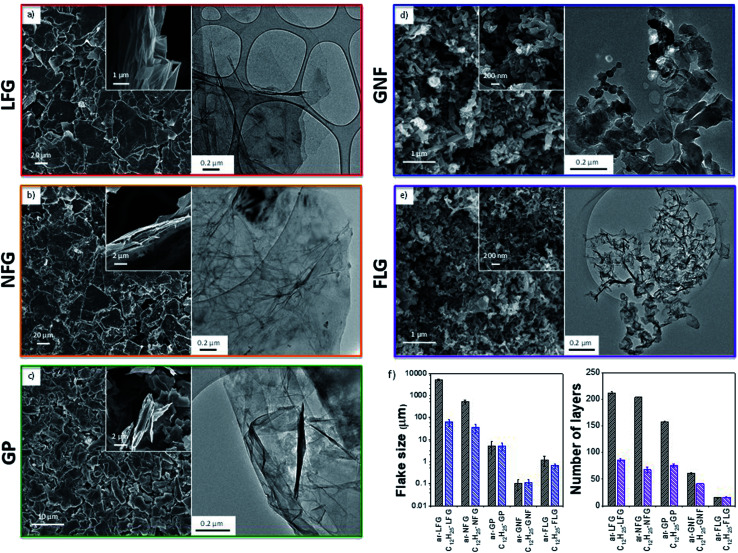
(a–e) SEM and TEM of the different type of graphite flakes after reductive treatment. (f) Comparison of average flake size (left panel) and number of layers (right panel) for as-received and functionalised graphites. Values obtained by SEM (LFG, NFG and GP), TEM (GNF) and AFM (FLG). Number of layers calculated from XRD.

**Scheme 1:**
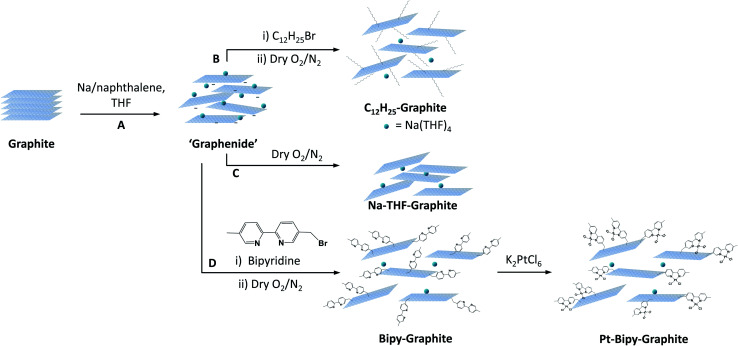
Generalised reaction scheme for dodecyl and 2,2′-bipyridine functionalisation of graphite. Reductive exfoliation (step A) followed by reaction with 1-bromododecane to yield dodecyl-functionalised graphite, C_12_H_25_-graphite (route B), direct quenching with dry O_2_/N_2_ to yield Na-THF-graphite (route C) or reaction with 5-monobromomethyl-5′-methyl-2,2′-bipyridine followed by single atom platinum deposition to yield Pt-bipy-graphite (route D).

Five representative starting graphites (large flake graphite (LFG), natural flake graphite (NFG), graphite platelets (GP), graphite nanofibres (GNF) and few-layer graphene (FLG)) with varying lateral sizes and morphologies (Fig. 1) were selected for this work. LFG and NFG are examples of crystalline graphites, whereas GNF and FLG consist of small stacks of graphene with much lower crystallinity. The GP sample lies at the interface between both groups of materials; it still retains the typical graphite crystallinity whilst displaying a larger degree of exfoliation when compared to LFG or NFG. The flake lateral size and the number of graphitic layers of the starting materials varied between 100 nm and 2 mm and between 15 and 220, respectively (Fig. 1 and Table 1). The exfoliation of the graphite starting materials was carried out using a standard methodology developed for grafting short alkyl groups.^1*a*,13^ Sodium and naphthalene were used as the reducing agent and transfer reagent, respectively. All graphite starting materials were exfoliated using a C/sodium ratio of 12; this ratio was previously found to be an optimum for graphene exfoliation/functionalization.^1*a*^ Each material was treated with sodium naphthalide in THF and functionalised with 1-bromododecane following route B (Scheme 1), as this functional group has a characteristic fragment for further characterisation using TGA-MS. The resulting dodecyl functionalisation is well explored, representing an excellent ‘model’ system, with only a moderate degree of steric occlusion.^1*a*,3^ The graphites were also functionalised with a methyl bipyridine molecule (route D, Scheme 1) followed by single atom platinum coordination. Functionalising the graphene layers with a tagging molecule that can complex heavy atoms, such as platinum, allows the functional group distribution to be studied using HAADF-STEM.

**Table 1:** Characterisation data for dodecyl-functionalised and bipy-functionalised graphites

Sample	Lateral size (μm)	Flake thickness^a^ (μm)	XRD layer number^b^	*I*_D_/*I*_G_	*I*_D_/*I*_D′_	*I*_2D_/*I*_G_	*n*_D_ (cm^−2^) × 10^11^	C/R (Raman)	GR (%)	C/R
ar-LFG	5000	50	212 ± 2	0.06 ± 0.05	1 ± 2	0.47 ± 0.07	9.2–7	5453–4152	—	—
C_12_H_25_-LFG	61 ± 21		86 ± 3	0.13 ± 0.17	2 ± 3	0.55 ± 0.18	21–16	2361–1797	1.1^c^	1246^c^
Bipy-LFG	61 ± 21			0.16 ± 0.12	—	0.24 ± 0.15	26–20	1915–1458	1.1^d^	983^d^
ar-NFG	500	10	204 ± 1	0.05 ± 0.05	1.3 ± 1.5	0.47 ± 0.04	8.8–6.7	5690–4332	—	—
C_12_H_25_-NFG	35 ± 16		68 ± 5	0.18 ± 0.18	3 ± 3	0.6 ± 0.2	30–23	1670–1272	1.7^c^	852^c^
Bipy-NFG	35 ± 16			0.17 ± 0.15	—	0.34 ± 0.05	28–21	1824–1388	2.5^d^	638^d^
ar-GP	5	0.5	157.8 ± 0.9	0.12 ± 0.02	3 ± 1.9	0.41 ± 0.02	19–15	2604–1983	—	—
C_12_H_25_-GP	5 ± 2		76 ± 3	0.48 ± 0.12	5.6 ± 1.9	0.5 ± 0.1	79–60	635–483	7.3^c^	193^c^
Bipy-GP	5 ± 2			0.17 ± 0.10	—	0.45 ± 0.04	28–21	1751–1333	19.5^d^	80^d^
ar-GNF	0.1	0.3	61 ± 1	1.56 ± 0.07	5.1 ± 0.4	0.37 ± 0.02	256–194	196–149	—	—
C_12_H_25_-GNF	0.11 ± 0.04		41.8 ± 0.7	1.7 ± 0.02	5.8 ± 0.6	0.45 ± 0.04	273–208	183–139	21.0^c^	67^c^
Bipy-GNF	0.11 ± 0.04			1.53 ± 0.06	—	0.37 ± 0.03	251–190	199–152	14.0^d^	111^d^
ar-FLG	1.2	0.02	15.2 ± 0.5	0.31 ± 0.04	1.1 ± 0.2	0.59 ± 0.07	51–39	988–752	—	—
C_12_H_25_-FLG	0.64 ± 0.17		16 ± 1	0.6 ± 0.2	4.8 ± 1.6	0.6 ± 0.1	105–80	475–362	25.7^c^	55^c^
Bipy-FLG	0.64 ± 0.17			0.49 ± 0.12	—	0.42 ± 0.1	80–61	625–476	23.2^d^	66^d^

a Obtained from AFM measurements.

b Obtained from XRD measurements.

c Obtained from TGA calculations.

d Obtained from XPS calculations.

The different graphites were all charged using sodium naphthalide, achieving a stable suspension of (partially) exfoliated graphitic material due to the coulombic repulsion between the charged layers. The dark green colour of the sodium naphthalide disappeared after addition to the graphites due to electron transfer from the naphthalide to the graphite feedstock. The charged graphites were either quenched with dry air for further characterisation or functionalised with either 1-bromododecane or the bromo-methyl bipyridine reagent. After treatment with 1-bromododecane, flake diameter generally decreased while the extent of exfoliation increased, as measured by SEM (LFG, NFG, GP), TEM (GNF) and AFM (FLG) (Table 1); the effects were more pronounced for the larger flakes (Fig. 1).

X-ray diffraction analysis (CuK_α_ = 1.542 Å) confirmed the greater graphitic character of the LFG, NFG and GP starting materials compared with GNF and FLG. A weakening and broadening in the graphitic (002) peak (2*θ* = 26.6°) was observed after functionalization for all the graphites, due to exfoliation during the reaction (Fig. S2†). The appearance of kinetically-trapped graphite intercalation compounds (GICs), which are more likely to remain in larger graphite flakes, complicates the interpretation.^14^ LFG and its derivatives show very similar diffraction patterns to NFG, with the (003) peak of a stage 1A Na-THF-GIC (2*θ* = 23.9°)^14,15^ present in the reductively exfoliated samples. For the smaller GP and GNF materials, reductive exfoliation results in a slight weakening of the graphite (002) peak. The (as-received) ar-FLG material shows a broad weak signal at 26.5°, corresponding to a layer spacing of 3.4 Å, which became broader after reductive exfoliation. Functionalization with dodecyl chains, in general, afforded further exfoliation for all the graphites. C_12_H_25_-LFG and C_12_H_25_-NFG both have a greatly reduced stage 1A (003) signal, and an increased random stage phase contribution to the graphite (002) peak, suggesting that alkyl chains prevent graphene sheets from fully restacking, allowing some diffusion of solvent molecules between the layers. For GP material, the graphite (002) peak is further weakened when grafted to form C_12_H_25_-GP. In the GNF materials, the weakened graphite (002) signal after exfoliation shows a further decrease after functionalization, and a large broad peak at ∼25.0° appears, corresponding to an interlayer spacing of 3.6 Å, which can be attributed to functionalized, imperfectly restacked graphene layers. Finally, for the FLG grafted sample, C_12_H_25_-FLG, very weak signals between 25° and 27° indicate that a high degree of exfoliation is retained, stabilized by the dodecyl chains. Overall, naturally occurring graphites (LFG and NFG) showed an increased exfoliation after charging but also evidence of trapped Na-THF intercalation, which disappeared when functionalized with dodecyl chains. GP, GNF and FLG also showed an increased exfoliation without retaining well-defined GIC compounds. Functionalization increased the degree of exfoliation for all types of materials.

**Fig. 2:**
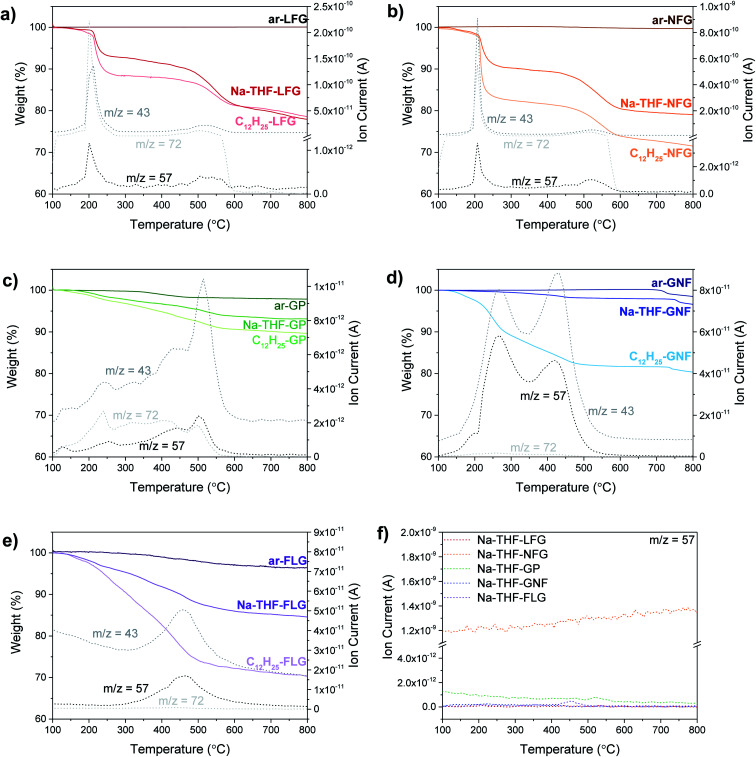
TGA-MS profiles of ar-, Na-THF, and C_12_H_25_- (a) LFG, (b) NFG, (c) GP, (d) GNF and (e) FLG; dodecyl and THF fragments *m*/*z* 57 (C_4_H_9_^+^), 43 (C_3_H_7_^+^/CHCH_2_O–^+^), 72 (C_4_H_8_O); (f) *m*/*z* 57 for each Na-THF-(graphite).

TGA-MS is a well-established method to quantify the degree of grafting. Although, the presence of trapped solvent can introduce additional mass losses, TGA-MS evidences the nature of the species evolving at different temperatures. Overall, larger graphites showed the presence of large amounts of trapped solvent and a lower degree of functionalization (LFG and NFG) whereas smaller graphites displayed the opposite behaviour (less trapped solvent and a larger degree of functionalisation, Fig. 2); the trends in trapped solvent are consistent with the XRD observations. More specifically, as a baseline, larger graphite starting materials (LFG, NFG and GP) showed no mass loss in the range from 100 °C to 800 °C, whereas smaller flake size graphites showed a small mass loss in this range (2.8 wt% for FLG and 1.5 wt% for GNF), probably due to the decomposition of oxygen functionalities. Exfoliated samples (Na-THF-graphite) showed a mass loss related to the presence of THF molecules (*m*/*z* 41, *m*/*z* 42) with a well-defined two-step weight loss for LFG, NFG and GNF. The two-step thermal degradation of the Na-THF intercalants may indicate the presence of two distinct solvent environments, or a partial decomposition of THF followed by further degradation of the remaining fragments.^14^ GP showed a continuous solvent loss in the range between 200 °C and 600 °C, with a more pronounced solvent peak at around 520 °C, possibly due to the formation of less defined solvent pockets within the layers. Surprisingly, GNF also showed a two-step mass loss despite its small flake size, most likely due to its graphitic character; although the degradation temperature of the second step was significantly lower (440 °C) than the one observed in the natural graphite sources (∼550 °C). The FLG material showed one single mass loss correlating with only one distinct THF peak from intercalated solvent-Na complexes as described previously;^16^ the small layer size is expected to promote solvent deintercalation.

All the graphites functionalised with 1-bromododecane (C_12_H_25_-graphite) showed larger mass losses compared to the Na-THF-graphites, regardless of flake size and morphology, with a mass fragment arising from dodecyl chains (*m*/*z* 57) clearly observed in each graphite material, confirming the grafting of dodecyl units to the graphene layers. Physisorption controls, mixing charged graphenes and dodecane, did not show any signals at *m*/*z* = 57 confirming that the work up removed unbound reagents (Fig. S3†). The grafting (weight) ratios and grafting stoichiometry C/R (both defined in ESI†) were estimated from analysis of solvent and dodecyl mass fragments and the accompanying mass loss in the corresponding temperature range following calculations reported previously (see ESI†).^17^ A general increase in grafting ratio, ranging from 1.1 wt% up to 25 wt%, was observed as flake size decreases (and defect density increases), corresponding to a decrease in C/R from 1200 down to 55 (Table 1). These trends are in agreement with the work reported by Hirsch, in which a decrease in C/R was observed when starting material defect density increased.^1*b*^

TGA values were not reliable for bipy-graphites as the typical fragments of bipyridine were not found in the MS analysis, possibly due to condensation of the heavier fragments before reaching the detector. However, due to the presence of a heteroatom in the bipyridine functional group, XPS was instead used to calculate grafting ratios and C/R values for this set of samples. TGA and XPS are both commonly used to report grafting ratios; the agreement between both techniques has been shown to be excellent in a recent review.^3^ In general, an increase in the grafting ratio was observed as flake size decreased, ranging from 1.1% up to 23.2%; corresponding to a decrease in C/R (from 983 down to 66). The XPS of the bipy-graphites, therefore, confirmed the functionalization of the graphites and the grafting ratios correlate closely with the TGA-MS values obtained for C_12_H_25_-graphites (Table 2).

**Table 2:** C/R and GR quantification data correlating values between graphites functionalised with C_12_H_25_ and bipyridine

Sample	Flake radius (μm)	GR (%) bipy^a^	GR (%) C_12_H_25_^b^	C/R bipy^a^	C/R C_12_H_25_^b^
LFG	30.5	1.1	1.1	983	1246
NFG	17.5	2.5	1.7	638	852
GP	2.5	19.5	7.3	80	193
GNF	0.05	14.0	21	111	67
FLG	0.32	23.2	25.7	66	55

a Obtained from XPS calculations.

b Obtained from TGA calculations.

**Fig. 3:**
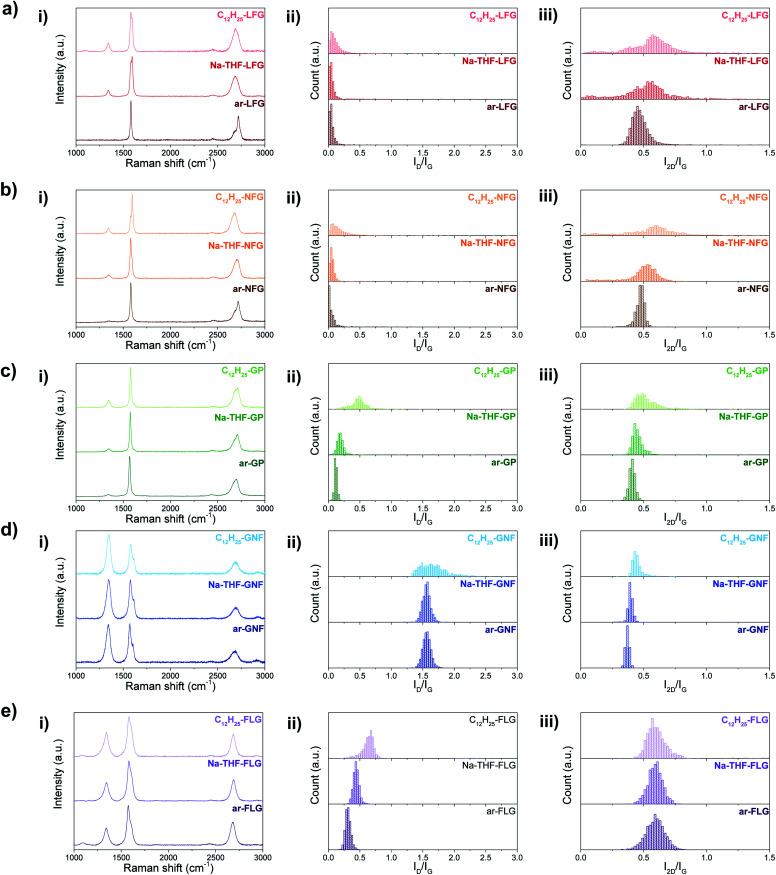
Averaged Raman spectra (i) and Raman histograms of *I*_D_/*I*_G_ (ii) and *I*_2D_/*I*_G_ (iii) ratio for ar-, Na-THF-, and C_12_H_25_- (a) LFG, (b) NFG, (c) GP, (d) GNF, and (e) FLG.

These grafting density trends are in good agreement with the statistical distributions of D to G band ratios (*I*_D_/*I*_G_) obtained from Raman measurements (Fig. 3); the large number of independent spectra fairly sample the materials present. The well-defined Raman spectra of the different graphites allow the classification of these materials in the ‘low defect density regime’,^18^ where the defect density does not exceed the Tuinstra–Koenig limit.^19^ Whereas LFG, NFG and GP starting materials showed a well-defined G band and a low intensity D band characteristic of low-defect graphitic structures, GNF and FLG showed a high intensity D band due to edge scattering and/or basal plane defects.^20^ However, there is a significant difference between both groups of graphites reflected in the presence of the D′ band in GNF starting material, indicating a larger amount of sp^3^ defects compared to edge-type defects.^9^ Overall, for the different selected graphites, with the exception of GNF, the *I*_D_/*I*_G_ ratio increases as the graphite layer size decreases, correlating with previously reported observations for different natural flake and spherical graphites (Table 1).^1*b*,21^ In principle, edge effects might manifest differently in the histograms, depending on whether the lateral flake size is larger or smaller than the laser spot size (590 nm), although none of the *I*_D_/*I*_G_ of larger flakes are significantly bimodal.

In the unfunctionalised, exfoliated Na-THF-graphite experiments LFG, NFG and GP showed no significant increase in *I*_D_/*I*_G_ ratio, confirming that the charging and quenching procedures result in no significant additional damage or inadvertent grafting on highly graphitic materials. FLG showed a slight increase in the *I*_D_/*I*_G_ ratio for the quenched material, suggesting that the process introduces some additional defects, potentially associated with edges or other pre-existing defects, as has been previously observed.^21,22^ GNF material did not show a significant increase in the *I*_D_/*I*_G_ ratio after exfoliation. After alkylation, the *I*_D_/*I*_G_ ratios increased for all the graphites, compared with the as-received and quenched samples, suggesting an increase in the number of sp^3^ atoms due to the presence of grafting sites;^1*a*^ this increase is most dramatic for the two smaller flake sizes which have the lowest C/R (highest degree of grafting). Histogram distributions slightly broadened for LFG and NFG, which had a low degree of functionalisation, whereas they significantly shifted and broadened for the rest of the graphite materials. A similar trend was observed for the bipyridine-functionalised graphites (Fig. S4†); however, the *I*_D_/*I*_G_ ratios in general were lower compared to the dodecyl functionalised graphites. *I*_2D_/*I*_G_ ratios were also lower in this case; one possible explanation for these lower ratios could be the contribution of the bipyridine group to an increase in the G band,^22,23^ resulting in apparently lower *I*_D_/*I*_G_ and *I*_2D_/*I*_G_ ratios, after normalisation.

**Fig. 4:**
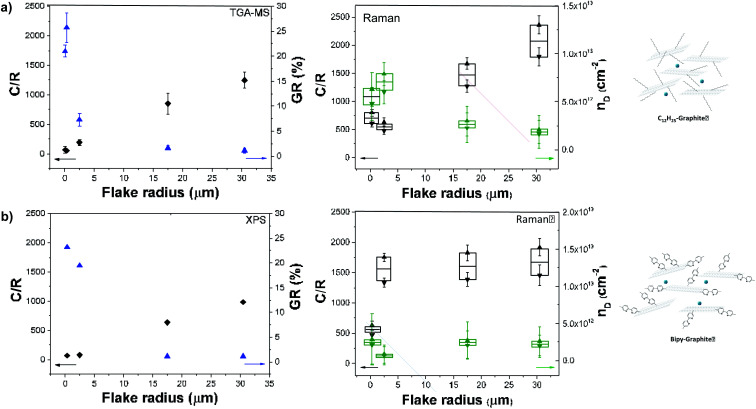
C/R (black) and GR (blue) obtained from TGA-MS data (left top panel) and XPS data (left bottom panel) and C/R (black) and *n*_D_ (green) obtained from Raman data (right panel) for (a) C_12_H_25_-graphites and (b) bipy-graphites.

The *I*_D_/*I*_G_ ratio can be used to estimate the inter-defect distance and number density of grafted sites per unit area (Table 1).^19^ The broadened histograms in the functionalised materials suggest that grafting may not occur uniformly (Fig. 3(ii)); therefore, the number density of grafted sites, *n*_D_, rather than interdefect distance, *L*_D_, is provided as the measure of grafting extent. A modified calculation of the conversion from *L*_D_ to *n*_D_ provided by Cançado *et al.*^19^ was used (ESI†), following similar calculations reported by Bepete *et al.*^24^ This model neglects the contribution of edges, thus the main origin of the D band is attributed to basal-plane defects. An estimate of the amount of defects can be obtained as a range, taking into account the structural radius of the defect (*r*_s_) and the activated radius (*r*_a_).^25^ In this way, we have obtained a lower and upper bound for the defect density and C/R values (Table 1, Fig. 4a and b, right panels). These estimates make a number of simplifications, including ignoring the effects of edge contributions, and the possibility that at higher degrees of functionalisation, the ‘activated’ areas overlap. Nevertheless, the estimated grafting densities are in the same range as the values obtained from TGA-MS and XPS calculations.

The grafting location on graphene sheets can have a significant effect on both interpretation of the chemistry and subsequent properties. Many previous discussions regarding reductive functionalisation have assumed homogeneous grafting across the flake,^1*b*,26^ with only a handful concluding that functionalisation is initiated by, and directed to, the edges.^1*c*,4^ In the case of homogeneous grafting across the basal surface of the graphene layer, the grafting ratio and C/R would be expected to remain constant as a function of flake size (between comparable starting graphites). In this grafting study, the grafting ratios obtained from TGA measurements and the grafting densities calculated from Raman *I*_D_/*I*_G_ vary inversely with flake radius (Fig. 4). A general correlation with flake size can be seen with grafting sites apparently scaling in relation to the available edges of each graphene layer, rather than the basal area, suggesting that edges might initiate and propagate grafting before inner sites (Fig. S1†). However, the overall degree of grafting (Table 1) is much too high (by up to two orders of magnitude) to be attributed to explicit edge sites only. The data also do not fit with grafting on the outer surfaces of the flake only; however, a close match is obtained assuming a band of grafting propagating inward on all layers (estimates provided in ESI, Table S1 and Fig. S5†).

In the case of fully exfoliated single-layer graphene, all carbon sites, edge and basal, are accessible to alkyl grafting; uniform functionalisation might therefore be expected. In intercalated graphite stacks where full exfoliation has not occurred, the inner basal planes are no longer as easily accessible, requiring diffusion of reactive moieties into the interlayer galleries before grafting can occur. In addition, grafted groups at flake edges may further inhibit functionalisation inside the flakes, due to the steric bulk of the chains; larger moieties would therefore be expected to enhance this effect, resulting in a functionalisation gradient, with a higher density of grafted sites propagating near flake edges (Fig. S1†). This model of a kinetically-favoured reaction near the flake edges that propagates inwards along the graphene basal plane is proposed here.

Further examination of Raman data, in particular the D and D′ bands, provides additional support for this mechanism (Fig. S6† and Table 1). In GNF, FLG, and GP samples, since the laser spot is similar or larger than the flakes, every spectrum is an average of all edge and sp^3^ contributions, and therefore the increase in *I*_D_/*I*_D′_, or sp^3^ to edge sites, can be straightforwardly taken as an indication of grafting. For NFG and LFG flakes, which are significantly larger than the laser spot, measurements show two distinct environments: edge and basal carbons. In the as-received materials, the larger ratio of basal to edge carbon sites results in a very low, practically zero, D band intensity, and a correspondingly low *I*_D_/*I*_D′_. The tail of the histogram to higher *I*_D_/*I*_D′_ is presumably derived from edge contributions. Following the proposed edge-initiated grafting, the *I*_D_/*I*_D′_ from the centre of the graphene flakes would remain unchanged, whilst the *I*_D_/*I*_D′_ at the edges would be expected to increase, as is indeed reflected in a lengthening of the tail of the histogram, seen in C_12_H_25_-LFG and C_12_H_25_-NFG, and absent in their as-received counterparts (Fig. S6†). On the other hand, if grafting were to occur uniformly across the flake, the whole histogram would be expected to shift to higher *I*_D_/*I*_D′_ values, reflecting the introduction of sp^3^ sites over the whole flake. The localisation implied in the Raman data is consistent with edge or defect-initiated grafting which is likely preferred over independent reactions on the relatively inert graphene basal plane. Hydrogenation of graphene has been proposed to initiate around a “nucleation point”, possibly a pre-existing defect, and then propagate to adjacent atoms due to the disruption of the sp^2^ network.^9,27,28^ While the steric bulk of the larger grafted groups, in this study, will prevent direct grafting on adjacent atom lattice sites, propagation has also been occasionally reported with bulkier groups, including during propagative reductive exfoliation of graphene.^29,30^ A previous study of Birch-type reduction of CNTs also showed that successive charging and functionalisation resulted in a propagative mechanism where functionalisation spread in bands over the tubes from previously grafted sites.^31^ Edge-propagation may be less relevant to carbon nanotubes (CNTs) since open ends are rarer, and the grafting is promoted by the tube curvature.^32^ However, flat graphene sheets are intrinsically less reactive, and hence the edges are more significant sites for initiation. In the current study, it is reasonable that grafting initiates at edges before propagating inwards through kinetic control.

In the larger, thicker natural graphites, this mechanism would be further favoured, since exfoliation is already energetically more difficult, and easy intercalation and diffusion of reactants inside the graphite is restricted,^4^ limiting reaction at inner sites. Further, natural defects may pin sheets together, meaning that only the outer surfaces and edges of the graphene layers are accessible for initiating and propagating functionalisation.

**Fig. 5:**
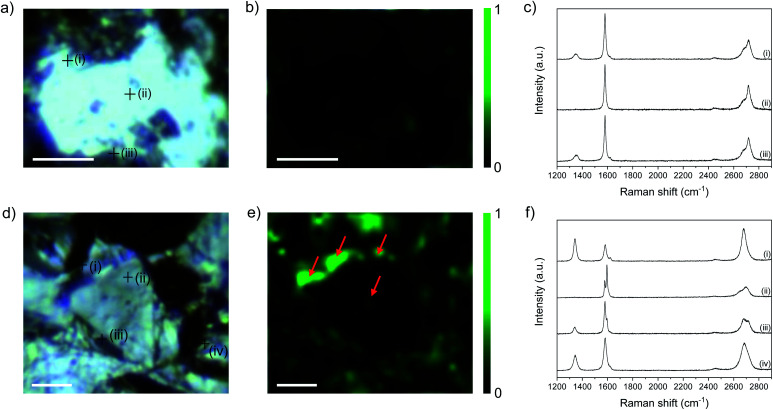
Comparison of flake edges in bath-sonicated NFG (top) and C_12_H_25_-NFG (bottom). (a and d) Optical micrographs, (b and e) Raman map of *I*_D_/*I*_G_, and (c and f) Raman spectra acquired at the positions marked in (a) and (d). Scale bar 10 μm.

Raman mapping provides additional evidence of edge-initiated functionalisation (Fig. 5) by comparing bath-sonicated NFG in ethanol (in order to obtain flakes with accessible edges (Fig. 5a–c)), and functionalised C_12_H_25_-NFG (Fig. 5d–f). NFG was selected for Raman mapping as it has the correct flake size, relative to the laser spot, to allow independent basal plane and edge imaging. In bath-sonicated NFG, the map of *I*_D_/*I*_G_ ratio illustrates the increased *I*_D_/*I*_G_ ratio around the edge of the flake, and at defect sites visible in the optical image. Example spectra taken at the points marked in (a) show a small D peak at edges, and none in the centre of the flake, as expected. In the functionalised graphite, C_12_H_25_-NFG, generally a much greater *I*_D_/*I*_G_ ratio can be seen (Fig. 5e), again concentrated around the edges of the finite width flake. The width of the bright regions (marked with an arrow) is larger than the laser spot size (590 nm), showing that there is a band of functionalisation beyond the explicit edge sites. The width of the feature, the variable edge orientation, and the number of layers in the flake, rules out a polarisation effect associated with edge type.^33^ In addition, a more pronounced D peak can be seen in point spectra taken at certain areas across the flake; these functionalities might have been introduced following propagation from pre-existing defects on the basal plane.

*I*_D_/*I*_D′_ ratios obtained from the representative spectra shown in Fig. 5c and f show a marked difference before and after functionalisation. In the control NFG spectra taken at the edges of the flake, the *I*_D_/*I*_D′_ is ∼4 (Table S4†), characteristic of edge-type defects; this value increases significantly to ∼10 in spectra taken from the edges of C_12_H_25_-NFG flakes, reflecting the sp^3^ defects introduced during grafting. Even in the area where only a small increase in the D band was observed (area (iii) in Fig. 5f), the *I*_D_/*I*_D′_ ratio indicates that covalent grafting did occur. The centre of the flake (area (ii) in Fig. 5f) did not show any significant *I*_D′_ contribution, correlating with the previous *I*_D_/*I*_G_ observations. Overall, the Raman maps show that the increase in *I*_D_/*I*_G_ is not just from additional exposure of flake edges following reductive treatment, but also from the introduction of sp^3^ defects, providing valuable evidence for localisation of grafting in the region near flake edges. 2D bands from both samples showed a very different pattern. Bath-sonicated NFG did not show a significant change in the 2D band from the starting material; the asymmetric shape of this band confirmed the lack of exfoliation of the material after sonication for all areas ((i)–(iii) Fig. 5c). On the other hand, spectra taken from the edges of C_12_H_25_-NFG flakes showed a symmetric and intense 2D band (areas (i) and (iv) Fig. 5f), confirming exfoliation of the material after functionalization. However, the 2D band remained asymmetric for inner layer areas (area (ii) Fig. 5f), probably due to a partial exfoliation of the material, again initiated from the edge.

**Fig. 6:**
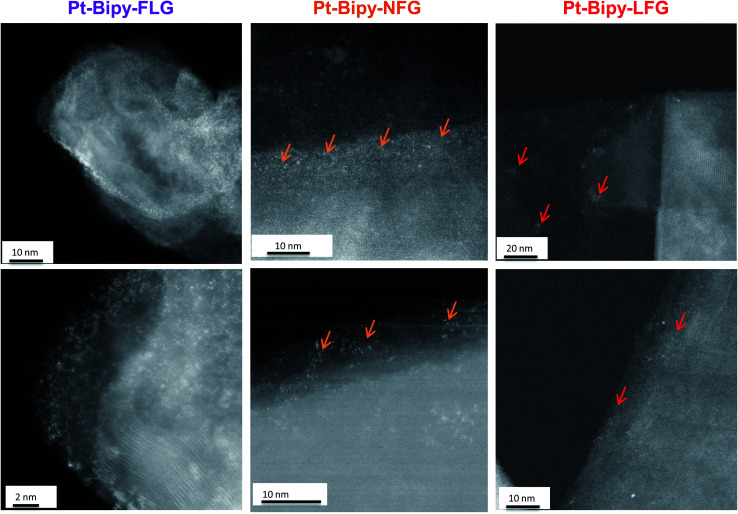
HAADF-STEM images of Pt-bipy-FLG (left panels), Pt-bipy-NFG (middle panels) and Pt-bipy-LFG (right panels). Orange and red arrows indicate the presence of platinum-bipyridine functional groups in clusters.

Further evidence of homogeneous (small flake size) or edge/cluster grafting (large flake size) was provided by complexing platinum single atoms as a tag to identify the locus of the functionalization on the bipy-graphites. Each platinum atom was coordinated to the bipyridine group and two chloride ligands (Scheme 1, route D). The materials were characterised using XPS (Fig. S7 and Table S2†). The N 1s component from XPS analysis provided proof of grafting for the bipyridine molecule (399.4 eV). After Pt was complexed to the bipyridine molecule, a new component was observed in the N 1s peak from the N–Pt contribution (400.5 eV) with a coordinated fraction of 79.3% and 70.1% for FLG and GP, respectively; for bipy-NFG and bipy-LFG samples, all the bipyridine rings were fully coordinated to Pt (Table S3†). The Pt 4f component provided further proof of the formation of the Pt complex: Pt(ii) 4f_5/2_ and Pt(ii) 4f_7/2_ components were observed in all cases (Fig. S7†). The absolute amount of platinum and nitrogen decreased as the lateral size of the graphene layer increased, in line with the grafting trends already discussed (Table 1 and Fig. 4b). The Pt XPS data are also consistent with the N XPS data for the uncomplexed bipy-large size graphites, affording one Pt atom per 2 N atoms (Table S2†). HAADF-STEM images of the as-prepared graphene samples evidence single-atom platinum coordination and provide insights into the locus of functionalisation (Fig. 6). Pt atoms were not observed in bright field mode (BF) (Fig. S8†); however, the Z-contrast in HAADF mode allowed the imaging of the high atomic number Pt species and revealed their distribution across the graphene layer. Medium size graphites (NFG) still showed a Pt distribution more localised along the edges of the layers, which is consistent with the NFG Raman mapping (Fig. 5) and with the kinetically-controlled edge or defect propagation model mentioned above; edge grafting was also observed for this material (Fig. 6, orange arrows). On the larger, more crystalline flakes (LFG) there was more evidence of functional group clustering on the basal plane although still located near the flake edges (Fig. 6, red arrows).

Grafting trends are distinctive for each graphitic material; smaller flakes (FLG) resulted in an upshift of *I*_D_/*I*_G_ and *I*_D_/*I*_D′_ ratios, suggesting a more uniform grafting across the graphene layers, as corroborated by the HAADF-STEM observations. On the other hand, larger layers showed a broadening in *I*_D_/*I*_G_ and *I*_D_/*I*_D′_ histograms, suggesting a heterogeneous grafting across the basal plane, most likely initiated by edges or by the presence of islands of defects across the basal plane. This heterogeneity is also observed in the HAADF-STEM images for NFG and LFG.

## 4. Conclusions

Reductive treatment with sodium naphthalide in THF was applied to a range of graphite materials with varying sizes and morphologies, to covalently graft dodecyl addends, which can be consistently identified in the TGA-MS, XPS and Raman data. Analysis of the Raman D and D′ bands confirms that the reduction itself does not cause additional damage to the carbon framework for large graphites; a slight increase in *I*_D_/*I*_G_ ratio was observed for smaller, more defective, FLG, but the effect was minor at this optimised charging ratio (Na : C 1 : 12).^22^ Damage may still occur if samples are charged excessively (for example at Na : C higher than 1 : 5).^34^ Crucially, the reductive functionalisation process improved the exfoliation of the material; graphene layers were stabilised by attachment of dodecyl addends, as reflected in the XRD and Raman data for the Na-THF-(graphite) and C_12_H_25_-graphite materials.

The flake size and morphology of the starting material were shown to affect the degree of grafting and the amount of residual solvent. Grafting of dodecyl and bipyridine addends provide useful model systems with which to study functionalisation parameters, especially in the larger flakes. A general increase in grafting ratio (ranging from 1.1% up to 25%) and a decrease in C/R, both extracted from TGA or XPS values, were observed as flake radius decreased. These C/R numbers were in reasonable agreement with values deduced from Raman statistical analysis. Further mapping using Raman spectroscopy of functionalised NFG illustrates that grafting is directed towards flake edges. This behaviour is further corroborated by HAADF-STEM of different graphite materials functionalised with single atom platinum complexes. Platinum was homogeneously distributed across small size layers (FLG); on the other hand, a more heterogeneous distribution with preferential edge grafting was observed for larger NFG and LFG materials. This mechanism of grafting is believed to arise from the relative inaccessibility of the inner basal plane to reactive moieties, resulting in preferential grafting at flake edges. The finite width of the functionalised region (of order 1 micron) means that small flakes are fully functionalised, to the extent that the size of the grafted addend allows, whilst larger flakes are functionalised more heterogeneously and to a much lesser extent on average. These findings might also be extrapolated to other related 2d materials. The prediction of graphene functionalization based on the initial morphology and size is very important when choosing the graphite starting material for a desired application. For example, small layer size and high functionalization density might be required for applications of graphene as electrocatalysts,^35^ where surface area and functional groups play a particularly important role. On the other hand, selective site functionalization might be of interest for applications of graphene in the sensing field.^36^ Moreover, single atom functionalised graphites may themselves find use in a wide range of applications, such as catalysts for hydrogen peroxide production or as electrocatalysts for oxygen reduction reaction.^11^

## Author contributions

The manuscript was written through contributions of all authors. All authors have given approval to the final version of the manuscript.

## Conflicts of interest

There are no conflicts to declare.

## Supplementary Material

SC-012-D1SC01958A-s001
