# Supplementary material for: Effect of graphene flake size on functionalisation: quantifying reaction extent and imaging locus with single Pt atom tags
Source: Chem Sci. 2021 Oct 21;12(44):14907–19. doi: 10.1039/d1sc01958a (PMC8597866; doi:10.1039/d1sc01958a)
Supplement: SC-012-D1SC01958A-s001 [file SC-012-D1SC01958A-s001.pdf]

Electronic Supplementary Material (ESI) for Chemical Science.
This journal is © The Royal Society of Chemistry 2021

Effect of Graphene Flake Size on Functionalisation: Quantifying Reaction Extent and Imaging Locus with Single Pt atom Tags

Noelia Rubio,‡1 Heather Au,‡1,2 Gabriel O. Coulter,[1] Laure Guetaz,[3] Gerard Gebel,[3] Cecilia Mattevi*4 and Milo S P Shaffer*1

[1]Departments of Chemistry & Materials, Imperial College London, London, UK.

[2]Department of Chemical Engineering, Imperial College London, London, UK.

[3]University Grenoble Alpes, CEA, LITEN, 38054 Grenoble Cedex 9, France.

[4]Department of Materials, Imperial College London, London, UK.

‡These authors contributed equally.

*Corresponding author.

*m.shaffer@imperial.ac.uk

Calculations of grafting density

The C/R ratio (the grafting density, or number of graphitic carbons per grafted moiety) was calculated from:

$$\mathrm{C/R} = \frac{MW_R}{wt\%_R} \times \frac{wt\%_C}{A_{rC}}$$

where MW_R and A_{rC} are the molecular weight of the grafted moiety and the atomic weight of carbon, respectively. The theoretical number density of grafting per cm^2, n_D, assuming perfect exfoliation, was obtained using the relation:

$$n_D\ (cm^{-2}) = \frac{10^{14}}{0.0262\ \mathrm{C/R}}$$

Where the denominator is the area of the graphene lattice per grafted moiety, calculated by multiplying the area occupied by one carbon atom (0.0262 nm^2) by C/R.

Calculations of number density of grafting (n_D) and interdefect distance (L_D) parameters

Raman I_D/I_G ratios were used to calculate number density of grafting, n_D, assuming a low density of defects below the Tuinstra-Koenig limit.[1] The original conversion from interdefect distance, L_D, to n_D uses L_D as the radius of the circle surrounding one defect;[2] in this work, L_D was calculated from the equation (following Bepete *et al*.[3]calculations):

$$L_D^2\ (cm^{-2}) = C_A \times \pi(r_A^2 - r_S^2)(I_D/I_G)^{-1}$$

$C_A = 160\ E_L^{-4}$, where E_L is the energy of the green laser (2.33 eV).

r_S is the structural size corresponding to the disordered area and r_A is the activated size of the area around the defect, as described by Luchese *et a.l*[4]. Bepete *et al* considered a possible range of 0.142 nm (carbon-carbon distance) and 0.5 nm (about 4 times this distance). These values were used in this work to calculate the L_D and n_D ranges.

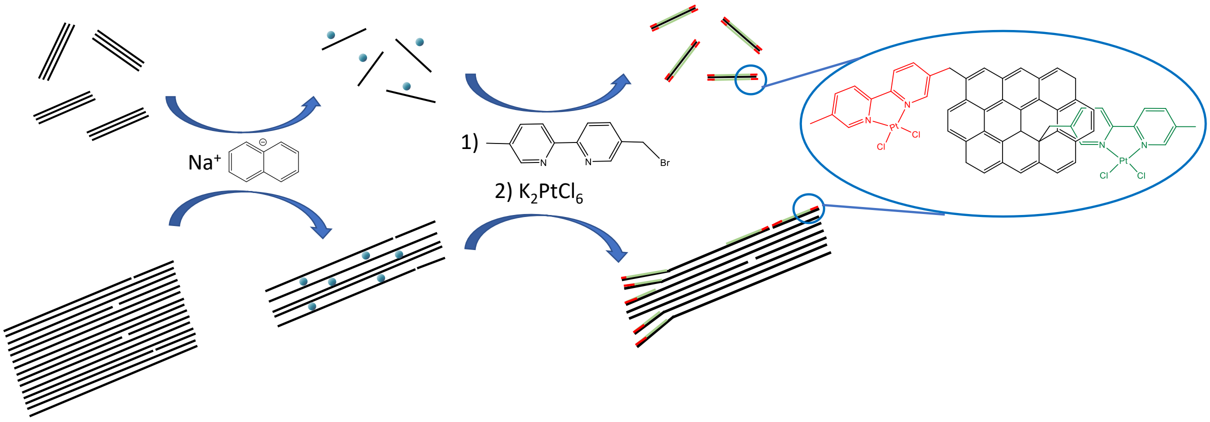

Figure S1. Schematic of the proposed GIC functionalisation following edge/defect-propagation model for small and large size graphite flakes.

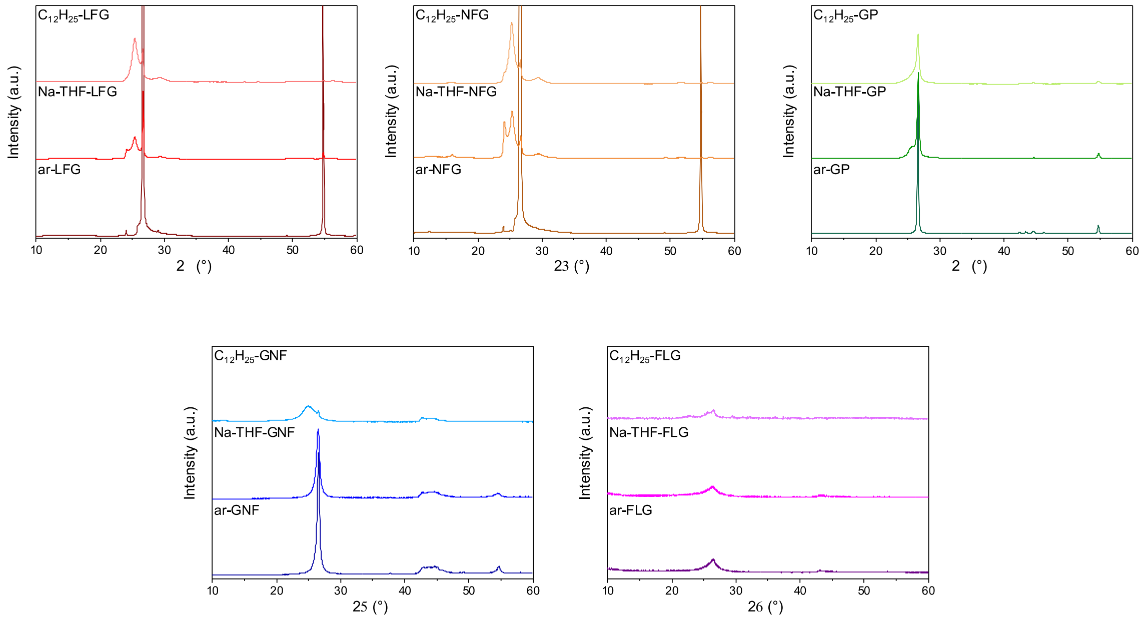

Figure S2. (i) XRD patterns for ar-, Na-THF- and $C_{12}H_{25}$-graphites. (ii) Raman histograms of I_{2D}/I_G ratio and Γ_{2D} (iii) for ar-, Na-THF-, and $C_{12}H_{25}$- a) LFG, b) NFG, c) GP, d) GNF, and e) FLG.

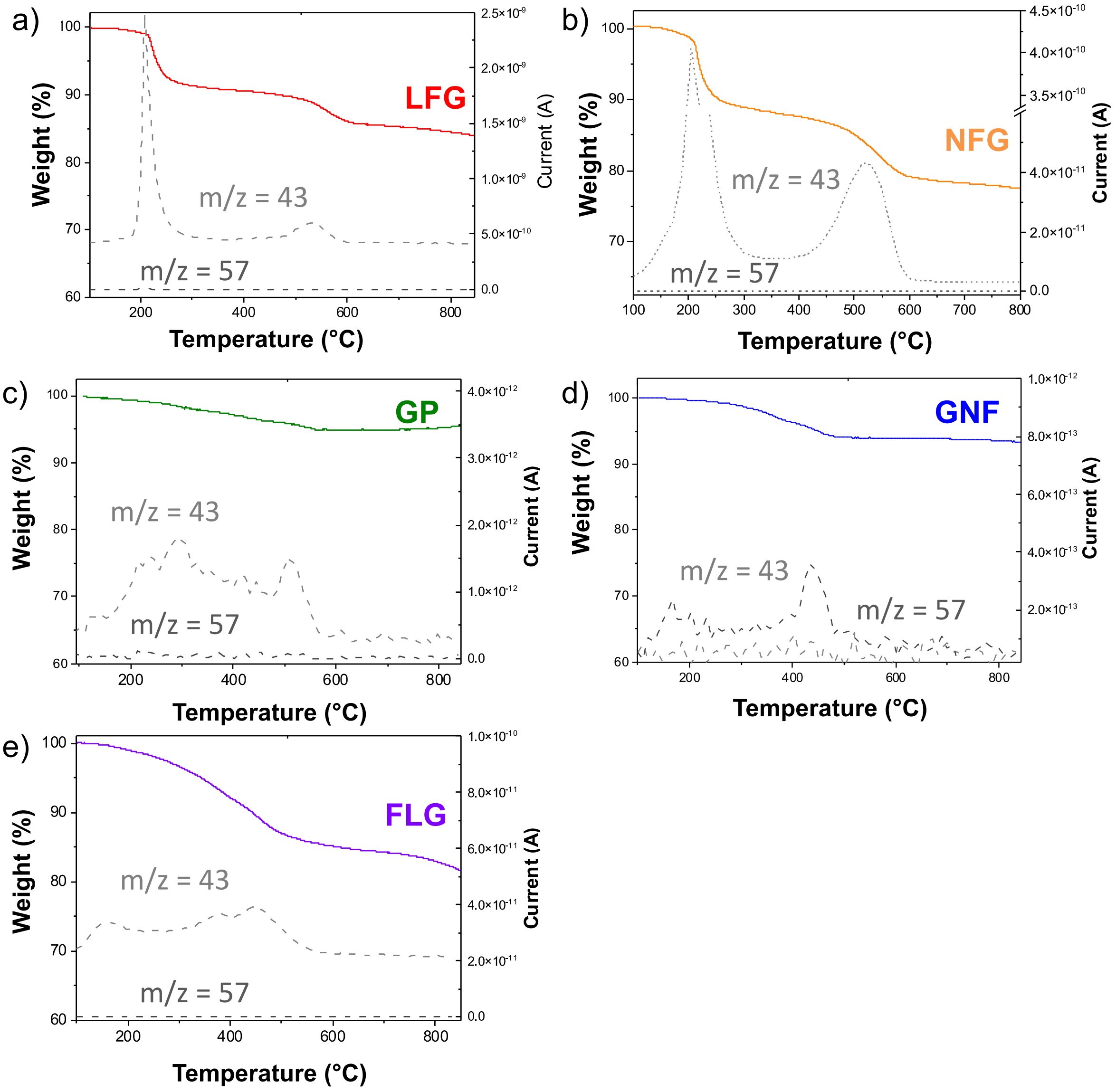

Figure S3. TGA-MS profiles of mixed control Na-THF-graphites and dodecane a) LFG, b) NFG, c) GP, d) GNF and e) FLG; dodecyl and THF fragments m/z 57 ($C_4H_9^+$), 43 ($C_3H_7^+$/$CHCH_2O$-$^+$) for each Na-THF-(graphite).

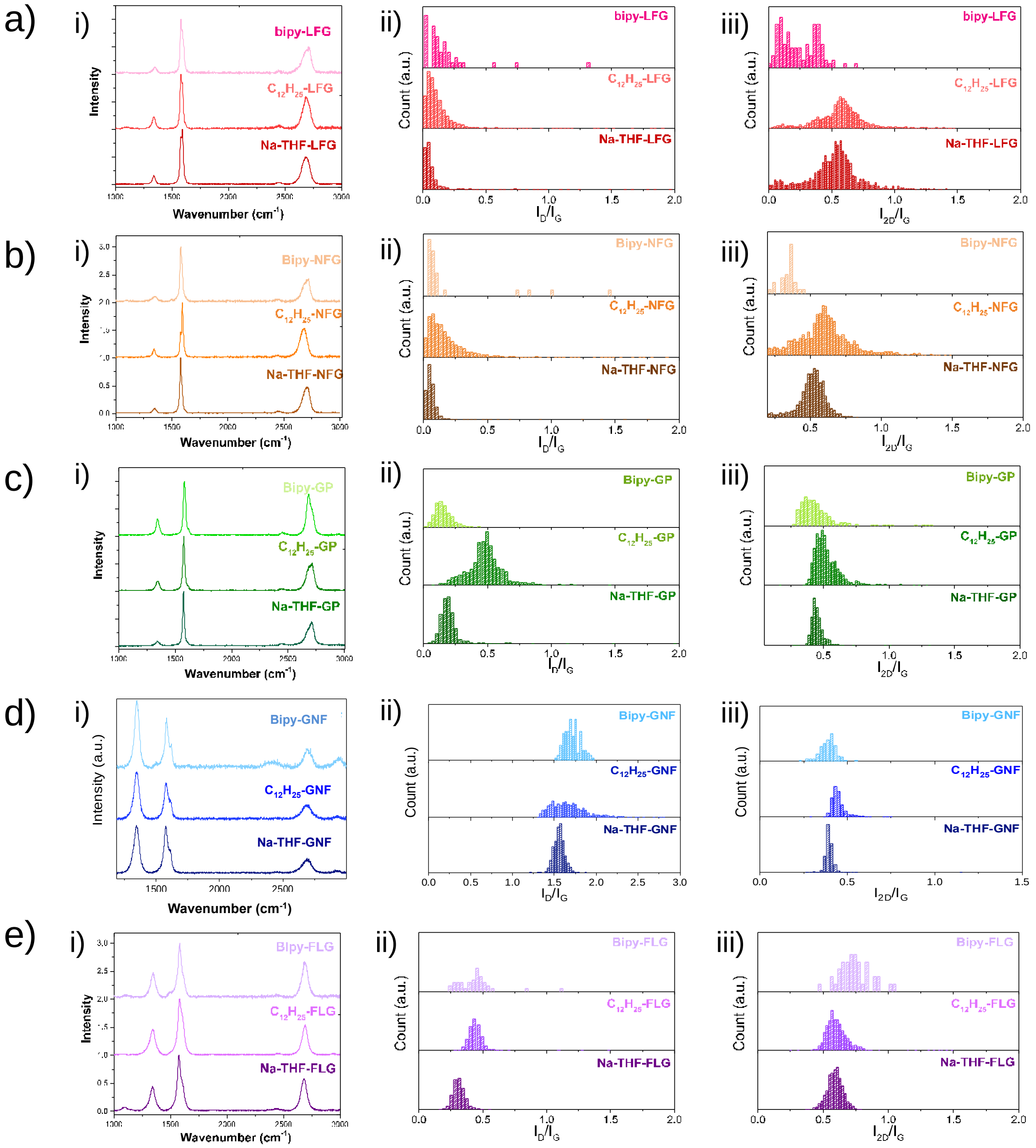

Figure S4. Averaged Raman spectra (i) and Raman histograms of I_D/I_G (ii) and I_{2D}/I_G (iii) ratio for Na-THF-, $C_{12}H_{25}$- and Bipy- a) LFG, b) NFG, c) GP and d) GNF and e) FLG.

Estimation of number of available reaction sites

Calculations to estimate the number of available reactions sites were made by considering the graphite platelets as a stack of *n* discs of graphene. The number of atoms in the plane was estimated from the 2d lattice of graphene (**a** = 0.246 nm, **γ** = 120°, 2 atoms per unit cell), giving 38.1 C atoms /nm^2. The number of atoms per unit length of edge was estimated as the average of the number of carbons per unit length of zig-zag edge (4.07 /nm) and armchair edge (4.69 /nm), ie 4.38 C atoms / nm. The fraction of edge carbons / total carbons (C_{edge}/C_{total}) was calculated straightforwardly from the average flake radius and number of layers in the stack, and is given in the table below (Table S1). The number of grafted chains (R/C) deduced in the manuscript [equivalent to R/C_{total}], can be renormalized to give R/C_{edge}. As seen in Table S1, there are around two orders of magnitude too many grafted chains to be attributed only to edge sites, for either the dodecyl or bipyridyl functionalisation; only the smallest (50 nm) radius flake is closer, but there are still insufficient edge sites to account for the degree of grafting. Any steric interaction between edge sites would only exacerbate the problem. Various models were applied to explain the possible distribution of grafted groups, including full and partial grafting on all external surfaces, but the only model that fitted well, was an edge-initiated functionalisation consistent with the microscopy shown in the paper (the "ring" model). This "ring" model assumes that all the edge sites are functionalized and then the reaction propagates inward on each layer to some characteristic length scale, or width (*W*). Within this planar region, the degree of functionalisation is limited by the steric interactions between the grafted groups. The steric limit is taken as R/C~60, from the flakes smaller than the characteristic width, such that all surfaces are functionalized; this value is consistent with previous estimates of steric limitations.[5] When the number of grafted chains (R) is normalized to the number of ring sites (C_{ring}), the resulting values of R/C_{ring} approach unity, with W = 0.5 µm, a value consistent with the observed microscopy (Figure 5, main manuscript); for flakes with radius smaller than W, it is assumed that the whole layer area is accessed. Thus, we propose the propagating band model outlined in the paper.

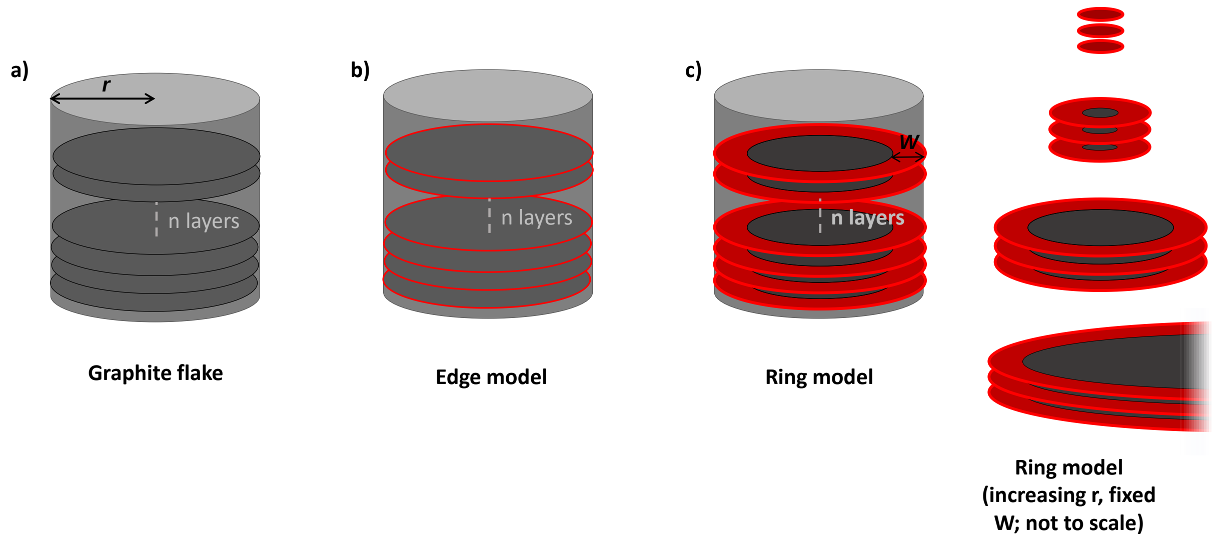

Figure S5. Representation of a graphite platelet where *r* is the flake radius and *n* the number of graphene discs in the stack (a). Representation of the edge model where edge functionalisation is shown in red (b). Representation of the ring model where ring functionalisation is shown in red and *W* is the propagation of the functionalisation.

Table S1. Calculated R/C values for both edge and ring models.

Sample	Flake radius (μm)	Layers per stack (n)	C/R Bipy	C/R $C_{12}H_{25}$	C_{edge}/C_{total}	C_{ring}/C_{total}	R/C_{edge} Bipy	R/C_{edge} $C_{12}H_{25}$	R/C_{ring} Bipy	R/C_{ring} $C_{12}H_{25}$
**LFG**	30.5	86	983	1246	7.52E-06	5.49E-04	135	107	1.9	1.5
**NFG**	17.5	68	638	852	1.31E-05	9.52E-04	120	90	1.6	1.2
**GP**	2.5	76	80	193	9.18E-05	6.09E-03	136	56	2.1	0.9
**GNF**	0.05	42	111	67	4.59E-03	2.13E-02	2	3	0.4	0.7
**FLG**	0.32	16	66	55	7.17E-04	1.74E-02	21	25	0.9	1.0

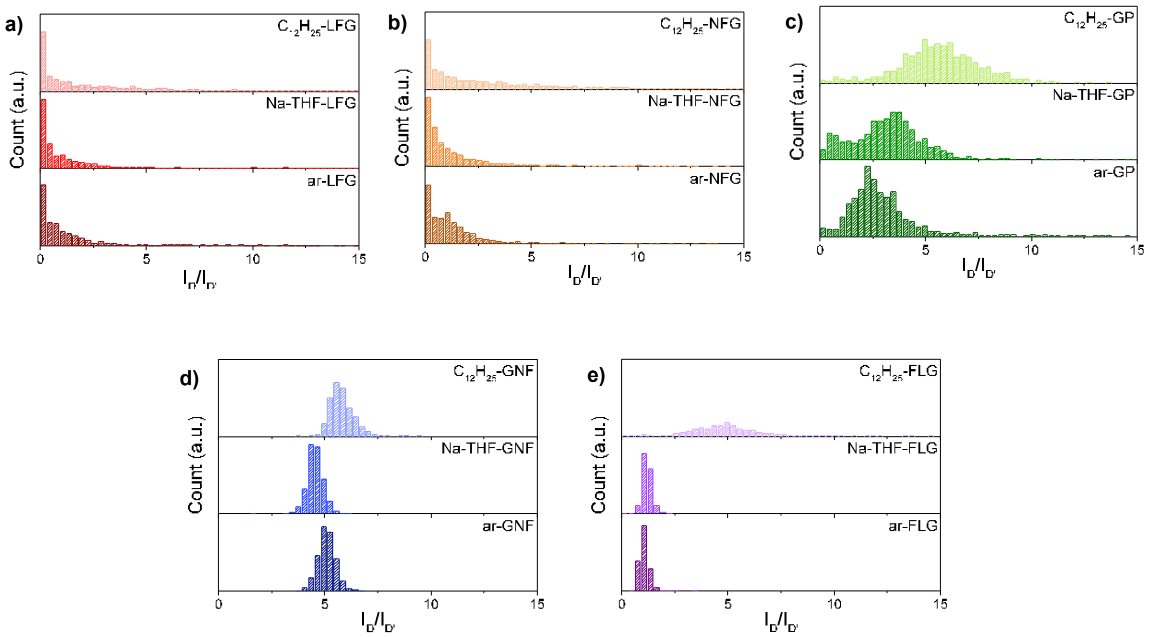

Figure S6. Raman histograms of $I_D/I_{D'}$ ratio for ar-, Na-THF-, and $C_{12}H_{25}$- a) LFG, b) NFG, c) GP, d) GNF, and e) FLG.

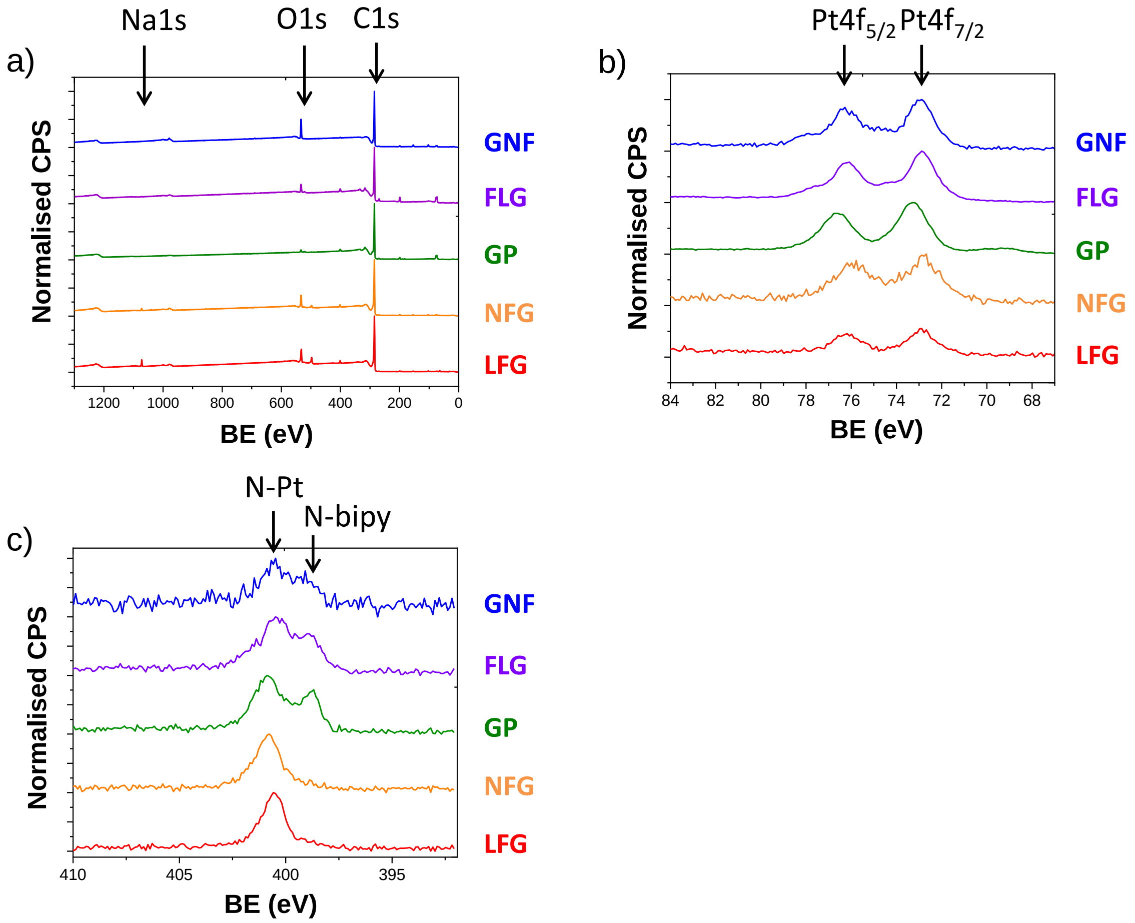

Figure S7. a) XPS surveys, b) Pt4f components and c) N1s components of the functionalised Bipy-Pt-graphites

Table S2. XPS quantification data of the different flake size graphites functionalised with platinum-bipyridine complex.

Sample	%C (at)	%N (at)	%O (at)	%Pt (at)	%Pt (mass)
**Bipy-Pt-GNF**	86.1	1.4	12.0	0.54	7.8
**Bipy-Pt-FLG**	89.9	3.5	5.4	1.3	17.1
**Bipy-Pt-GP**	95.3	2.4	1.6	0.73	10.7
**Bipy-Pt-NFG**	92.5	0.3	6.7	0.14	2.2
**Bipy-Pt-LFG**	90.3	0.15	7.3	0.06	0.98

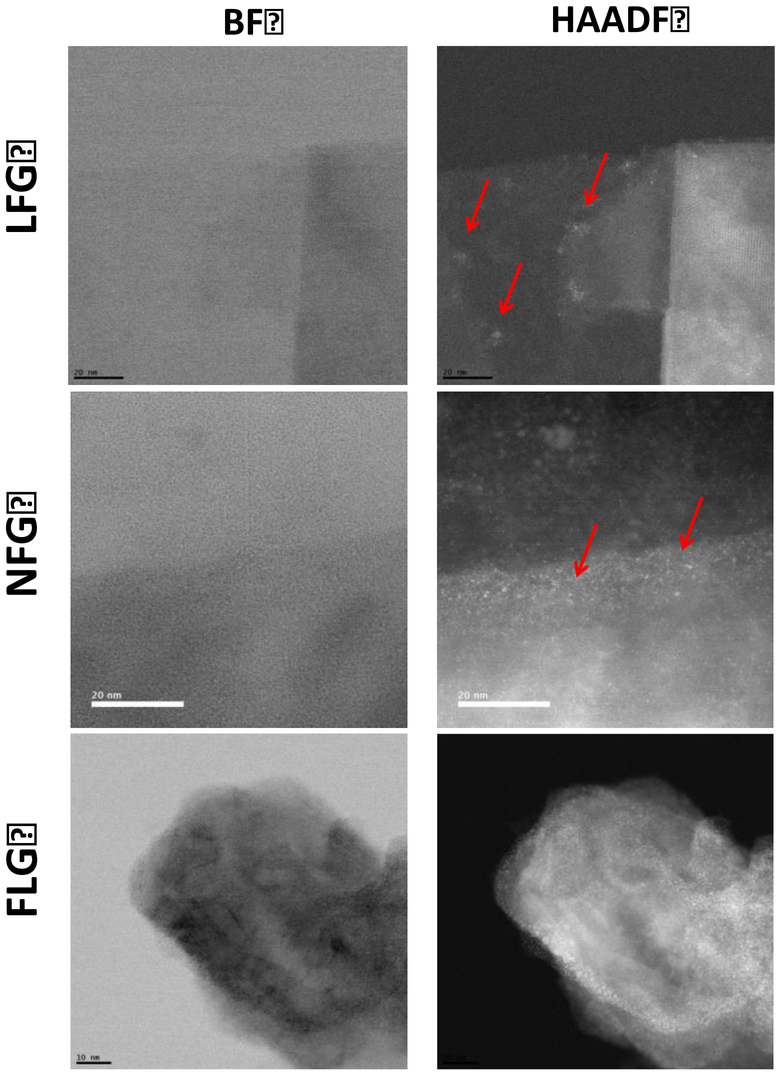

Figure S8. STEM images in BF (left panels) and HAADF mode (right panels) of Pt-Bipy-LFG, Pt-Bipy-NFG and Pt-Bipy-FLG. Red arrows indicate the presence of platinum-bipyridine functional groups in clusters in HAADF mode.

Table S3. Contributions of coordinated Pt to bipyridine (N-Pt) and unbound nitrogen to N1s component.

Sample	N-Pt	Unbound N
**Bipy-Pt-GNF**	76.6	23.4
**Bipy-Pt-FLG**	79.3	20.7
**Bipy-Pt-GP**	70.1	29.9
**Bipy-Pt-NFG**	100	-
**Bipy-Pt-LFG**	100	-

Table S4. Summary of I_D/I_G and $I_D/I_{D'}$ values of the spectra obtained in Figure 5.

Spectrum	I_D/I_G	$I_D/I_{D'}$
**NFG (i)**	0.12	5.04
**NFG (ii)**	0	-
**NFG (iii)**	0.13	2.98
**$C_{12}H_{25}$-NFG (i)**	1.37	9.67
**$C_{12}H_{25}$-NFG (ii)**	0	-
**$C_{12}H_{25}$-NFG (iii)**	0.21	10.38
**$C_{12}H_{25}$-NFG (iv)**	0.47	9.43

[1] F. Tuinstra, J. L. Koenig, *J Chem Phys* **1970**, *53*, 1126-&.

[2] L. G. Cancado, A. Jorio, E. H. M. Ferreira, F. Stavale, C. A. Achete, R. B. Capaz, M. V. O. Moutinho, A. Lombardo, T. S. Kulmala, A. C. Ferrari, *Nano Lett* **2011**, *11*, 3190-3196.

[3] G. Bepete, A. Penicaud, C. Drummond, E. Anglaret, *J Phys Chem C* **2016**, *120*, 28204-28214.

[4] M. M. Lucchese, F. Stavale, E. H. M. Ferreira, C. Vilani, M. V. O. Moutinho, R. B. Capaz, C. A. Achete, A. Jorio, *Carbon* **2010**, *48*, 1592-1597.

[5] A. J. Clancy, H. Au, N. Rubio, G. O. Coulter, M. S. P. Shaffer, Dalton Trans., 2020, 49, 10308-10318
